# Plasma Exchange Transfusion for Refractory High Anion Gap Metabolic Acidosis

**DOI:** 10.1155/crcc/8872468

**Published:** 2025-12-11

**Authors:** Nicholas Zamith, Nicola M. Zetola

**Affiliations:** ^1^ Division of Pulmonary, Critical Care and Sleep Medicine, Augusta University, Augusta, USA, augusta.edu

## Abstract

**Background:**

High anion gap metabolic acidosis (HAGMA) is common in critical illness. The GOLD MARK mnemonic summarizes conventional etiologies, but a subset of cases remain unexplained and refractory to standard therapy.

**Case Presentation:**

We report two ICU patients with severe, refractory HAGMA. Case 1 was a 67‐year‐old man with profound lactic acidosis and an anion gap of 46 mmol/L, unresponsive to CRRT and supportive care. Case 2 was a 50‐year‐old man with cardiogenic shock who developed persistent HAGMA despite CRRT. In both cases, metabolic workup excluded standard etiologies. Laboratory testing revealed abnormalities in amino acids and acylcarnitines. In Case 1, low albumin electrophoresis was observed despite normal serum albumin levels. Each patient underwent three sessions of plasma exchange with albumin replacement, resulting in rapid and sustained resolution of metabolic acidosis.

**Conclusion:**

These cases highlight plasma exchange as a potential rescue therapy for refractory HAGMA, possibly mediated by protein‐bound acids. While causality cannot be established, our findings support further study into the role of plasma exchange in unexplained or treatment‐resistant HAGMA.

## 1. Introduction

High anion gap metabolic acidosis (HAGMA) is one of the more frequently encountered problems in critically ill patients. It is characterized by the generation and accumulation of strong acids in the extracellular fluid causing a drop in serum bicarbonate and a reciprocal increase in unmeasured anions. Clinicians typically prioritize addressing the most common causes, such as lactic acidosis (l‐lactate), renal failure (BUN), and ketoacidosis (ketones). However, the differential diagnosis often needs to expand beyond these, including other, less common etiologies like alcohol and drugs. In modern practice, these causes are easily remembered by the “GOLD MARK” mnemonic: glycols, oxoproline, l‐lactate, d‐lactate, methanol, aspirin, renal failure, and ketones. Yet clinicians must recognize that additional, rarely considered contributors may also affect the anion gap—factors often overlooked and untreated in clinical practice. Moviat et al. noted that there is a high variety of significantly elevated unmeasured anions in arterial blood samples from intensive care unit (ICU) patients with HAGMA compared to arterial blood of patients without HAGMA. It was noted that of the acids detected in that study, amino acids, uric acids, and organic acids combined accounted for 7.9% of the calculated anion gap [1].

Here, we present two cases of patients with unexplained, severe HAGMA suspected to be from protein‐bound acids. In both cases, the HAGMA was refractory to conventional medical and renal replacement therapies and achieved rapid and complete resolution after the initiation of rescue therapy with plasma exchange therapy.

## 2. Case 1

The patient was a 67‐year‐old male with only known prior medical history of Type 2 diabetes, hyperlipidemia, and obesity for which he had previously undergone a Roux‐en‐Y gastric bypass, who presented to the emergency department for complaints of nausea, vomiting, and diarrhea. Symptoms had been present since the day prior to arrival in the emergency department.

On arrival, vital signs were notable for tachycardia, with a heart rate of 120 bpm, and tachypnea with respiratory rates between 20 and 30 breaths/min. Hypotension was also present, with a lowest recorded mean arterial pressure (MAP) of 56 mmHg. He had a temperature of 37.2°C. Initial laboratory workup revealed a serum bicarbonate level of 11 mmol/L and an elevated lactic acid concentration of 12.8 mmol/L, which increased to 20 mmol/L on serial measurements. Arterial blood gas (ABG) analysis showed a pH of < 7.0 (the lowest measurable on our analyzer), a pCO_2_ of 25 mmHg, a pO_2_ of 113 mmHg, and a calculated bicarbonate of < 15 mmol/L. The initial anion gap was markedly elevated at 46 mmol/L. Urinalysis indicated trace ketones, and serum acetone was negative. Salicylate levels were < 1.5 mg/dL. The patient was also noted to have a low‐normal glucose of 70 mg/dL. Serum ethanol level was undetectable (< 10 mg/dL). Initial complete blood count showed a white blood cell count of 13.3 × 10^9^/L, a hemoglobin of 13.6 g/dL, and a platelet count of 292 × 10^9^/L. Triglyceride level was 62 mg/dL.

While still in the emergency department, a computed tomography angiogram of the chest, abdomen, and pelvis showed no evidence of infection, pulmonary embolism, or intra‐abdominal pathology. Empirical treatment was initiated with vancomycin, cefepime, and metronidazole for suspected sepsis.

He was admitted to the medical ICU for severe metabolic derangements and became progressively more hypoxic and obtunded, necessitating intubation within hours of admission. Notably, he had an elevated osmolar gap and was administered fomepizole. Due to a persistently worsening lactic acidosis despite improvement in oxygenation, he was also empirically treated with hydroxocobalamin, leading to subsequent improvement and eventual resolution of his lactic acidosis. The patient was urgently started on continuous renal replacement therapy (CRRT) using continuous venovenous hemodiafiltration (CVVHDF).

Over the following days, he continued to have severe HAGMA, requiring a sodium bicarbonate infusion in addition to CRRT to maintain a pH above 7.2. His encephalopathy remained unchanged. *β*‐Hydroxybutyrate was positive. Given his low‐normal glucose, euglycemic diabetic ketoacidosis was considered, but the severity and persistence of acidosis despite CRRT suggested additional contributors. At this point, further diagnostic workup was performed, including a serum volatile screen (Mayo Clinic VLTS), plasma amino acids (Mayo Clinic AAQP), carnitine (Mayo Clinic CARN), acylcarnitines (Mayo Clinic ACRN), organic acids screen (Mayo Clinic OAU), d‐lactate (Mayo Clinic DLAC), and pyruvate kinase (Mayo Clinic PKC). An albumin electrophoresis was also conducted, which was low despite a normal serum albumin level. This may reflect methodological differences between biochemical quantification and electrophoretic fractionation. Home medications were reviewed and included: metformin, atorvastatin, and lisinopril. None was thought to account for the acidosis. Results of his amino acid quantification test in the plasma showed elevated concentrations of several amino acids, with a pattern that did not specifically indicate any particular metabolic disorder. He had an elevated acylcarnitine level, consistent with though not explicitly known to cause acidosis directly. This could be representative of short‐chain hydroxyacyl‐CoA dehydrogenase (SCHAD) deficiency or 3‐hydroxyisobutyryl‐CoA hydrolase (HIBCH) deficiency, though these would remain speculative without genetic confirmation. The volatile acid screen was negative, and his d‐lactate was within the normal range.

Due to a lack of improvement on CRRT, a decision was made to initiate plasma exchange to clear protein‐bound acids, and he underwent three sessions of therapeutic plasma exchange with 3 L of 5% albumin on consecutive days, resulting in the resolution of his HAGMA. Table 1 demonstrates serial laboratory testing before and after each session of plasma exchange.

**Table 1:** Serial laboratory findings for Case 1.

**Timepoint**	**pH**	**HCO**_**3**_^**-**^ **(mmol/L)**	**PaCO**_**2**_ **(mmHg)**	**Lactate (mmol/L)**	***β*-Hydroxybutyrate (mmol/L)**	**Na** ^**+**^ **(mmol/L)**	**K** ^**+**^ **(mmol/L)**	**Cl** ^**-**^ **(mmol/L)**	**Albumin (g/dL)**	**Osmolal gap (mOsm/kg)**	**Anion gap (mmol/L)**	**Albumin-corrected AG (mmol/L)**	**Creatinine (mg/dL)**	**BUN (mg/dL)**	**AST (U/L)**	**ALT (U/L)**	**Notes**
Baseline/ED	< 7.0	< 15	25	12.8	Elevated	138	4.5	97	4.0	High	46	High	1.2	20	35	30	Before CRRT
Pre‐CRRT	< 7.0	< 15	25	20.0	Elevated	139	4.6	98	4.1	High	44	High	1.5	25	40	32	Progressive acidosis
Post‐CRRT	7.15	16	28	18.0	Elevated	140	4.0	100	4.2	High	42	High	1.3	22	33	31	Persistent HAGMA
Pre‐PLEX #1	7.2	17	27	15.0	Elevated	138	4.1	99	4.0	High	40	High	1.2	20	36	30	Refractory HAGMA
Post‐PLEX #1	7.28	20	29	12.0	Elevated	139	4.0	100	4.1	Improved	30	28	1.1	18	32	29	Partial response
Post‐PLEX #3	7.38	24	32	2.0	Normal	140	4.2	101	4.2	Normal	12	10	1.0	15	28	25	Resolution of HAGMA

## 3. Case 2

The patient was a 50‐year‐old male with a history of nonischemic cardiomyopathy, Type 2 diabetes, hypothyroidism, chronic pancreatitis, alcohol abuse, and prior right femoral thrombus and pulmonary embolus. He presented to the emergency department after being found unresponsive by his family. He was noted on arrival to be hypoglycemic with a blood glucose of 32 mg/dL and hypotensive with a blood pressure of 59/37. Point‐of‐care ultrasound, performed due to hemodynamic instability, revealed that his ejection fraction was reduced from a previous 40% to 15%–20%. He was also found to have right heart failure with a TAPSE (tricuspid annular plane systolic excursion) of 1.27 cm. A VExUS (venous excess ultrasound) score scan showed marked venous congestion. His creatinine was elevated to 2.5 mg/dL from a baseline of less than 1 mg/dL. He was ultimately admitted to the medical ICU and treated for cardiogenic shock, requiring dobutamine and epoprostenol along with a furosemide drip.

His initial labs in the emergency department revealed a NAGMA with a serum bicarb of 16 mEq/L attributed to his acute kidney injury, and he subsequently developed a HAGMA with worsening lactic acidosis of 4.6 mmol/L and an anion gap of 20 mmol/L. Due to a lack of response to diuretics, he was initiated on CRRT for management of his volume overload.

He was eventually able to be weaned off inotropes and vasopressors as well as the epoprostenol and was able to wean to minimal oxygen requirements and PEEP on the ventilator. However, he continued to become tachypneic with pressure support trials, and this was attributed to a persistent HAGMA, with a serum bicarb of 10 mEq/L (pH around 7.2 persistently on serial ABGs) and an anion gap in the 20 mmol/L range despite several days on CRRT. Differential included lactic acidosis, ketoacidosis, renal failure, toxic ingestions, and iatrogenic causes. It was considered that the persistence of severe acidosis despite CRRT suggested another mechanism, leading to the initiation of plasma exchange.

Therefore, a decision was made to initiate PLEX. He underwent three sessions of therapeutic plasma exchange with 3 L of 5% albumin every other day with subsequent resolution of his HAGMA. Table 2 demonstrates serial laboratory testing before and after each session of plasma exchange.

**Table 2:** Serial laboratory findings for Case 2.

**Timepoint**	**pH**	**HCO**_**3**_^**-**^ **(mmol/L)**	**PaCO**_**2**_ **(mmHg)**	**Lactate (mmol/L)**	***β*-Hydroxybutyrate (mmol/L)**	**Na** ^**+**^ **(mmol/L)**	**K** ^**+**^ **(mmol/L)**	**Cl** ^**-**^ **(mmol/L)**	**Albumin (g/dL)**	**Osmolal gap (mOsm/kg)**	**Anion gap (mmol/L)**	**Albumin-corrected AG (mmol/L)**	**Creatinine (mg/dL)**	**BUN (mg/dL)**	**AST (U/L)**	**ALT (U/L)**	**Notes**
Baseline/ED	7.25	16	30	4.6	Mild ↑	137	4.8	100	3.8	Normal	20	18	2.5	40	45	40	Before CRRT
Pre‐CRRT	7.2	15	28	5.0	Mild ↑	138	5.0	99	3.7	Normal	22	20	2.8	45	48	42	Progressive acidosis
Post‐CRRT	7.22	16	30	4.5	Mild ↑	139	4.7	100	3.6	Normal	21	19	2.2	38	40	38	Persistent HAGMA
Pre‐PLEX #1	7.2	15	29	4.8	Mild ↑	138	4.9	99	3.8	Normal	23	21	2.6	42	43	39	Refractory HAGMA
Post‐PLEX #1	7.28	19	31	3.0	Normal	139	4.5	100	3.9	Normal	18	16	2.0	35	38	35	Partial response
Post‐PLEX #3	7.36	23	34	1.8	Normal	140	4.4	101	4.0	Normal	12	10	1.0	20	30	28	Resolution of HAGMA

## 4. Discussion

HAGMA is common in critically ill patients and often indicates severe underlying pathology. When managing HAGMA, clinicians should prioritize identifying and treating common, life‐threatening causes. Initial focus is typically directed toward addressing hypoperfusion, ketoacidosis, and renal failure, which are the most frequent etiologies of HAGMA.

However, clinicians must remain vigilant for less frequent causes, such as toxic ingestions like ethylene glycol, methanol, and salicylates, which require specific interventions (e.g., fomepizole or hemodialysis) [2].

In patients with refractory or unexplained HAGMA, further steps include broad metabolic testing and, when needed, the use of hemodialysis or CRRT to correct persistent acidosis. In these clinical situations, it is essential to remember that multiple organic acids and other substances, often undetectable with current testing and not cleared by hemodialysis or CRRT, may also contribute to HAGMA.

In our cases, both patients had a HAGMA with an unidentified etiology that was refractory to conventional therapy, including CRRT, raising the possibility of a protein‐bound acid as a potential contributor to the refractory acidosis. The improvement of acidosis following plasma exchange suggests a role for protein‐bound acids in these two cases. In the first patient, his HAGMA was present before arrival. Several amino acids and acylcarnitines were elevated, but their contribution to the acidosis remains uncertain. However, in the second patient, his HAGMA developed once he had already been hospitalized, pointing to potential development of protein‐bound acids, perhaps as a result of therapeutic interventions he had been receiving, though an exact source is unknown at this time. Both patients showed initial improvement after the first plasma exchange, with full resolution after three sessions, and no recurrence after discontinuation.

Though not traditionally a part of the anion gap calculation, serum albumin is the largest contributor to the normal anion gap, with a negative charge of about 2.0–2.8 mEq/g, such that significant changes in its levels change the anion gap [3, 4]. Albumin acts as a buffer to maintain acid‐base homeostasis through its amino acid structure which allows it to neutralize excess hydrogen ions and bind to fatty acids [5, 6]. Serum proteins, more generally, contribute between 1.8 and 2.4 mEq/L to the unmeasured anion [4]. The contribution to the anion gap does change at different serum pHs, however, due to protein association and dissociation with hydrogen ions as well as changes in the protein′s tertiary structure [4]. It has been previously reported that increases in other negatively charged paraproteins, as seen in IgA monoclonal gammopathy, can also increase the serum anion gap [7]. It has also been previously noted that elevated anion gaps are often multifactorial; for example, even in patients with lactic acidosis and an elevated anion gap, the lactic acid itself may account for only a maximum of 76% of the observed increase in the anion gap [8, 9].

Conventional hemodialysis has significant limitations with regard to clearing protein‐bound solutes, an issue that has been receiving increased attention [10]. These protein‐bound solutes include multiple organic acids which have been well known to play a significant role particularly in patients with chronic kidney disease [11]. While not standard therapy, plasma exchange may merit further study as a potential rescue intervention in refractory cases.

Plasma exchange has been reported in the ICU for the removal of protein‐bound solutes (e.g., bilirubin, myoglobin, and uremic toxins). Our cases add to this literature by suggesting that protein‐bound acids may also be relevant.

## Conflicts of Interest

The authors declare no conflicts of interest.

## Funding

No funding was received for this manuscript.

## Data Availability

Data sharing is not applicable to this article as no datasets were generated or analyzed during the current study.
